# Autism Spectrum Disorder: Why Do We Know So Little?

**DOI:** 10.3389/fneur.2018.00670

**Published:** 2018-08-17

**Authors:** Leonardo Emberti Gialloreti, Paolo Curatolo

**Affiliations:** ^1^Department of Biomedicine and Prevention, Tor Vergata University of Rome, Rome, Italy; ^2^Child Neurology and Psychiatry Unit, Systems Medicine Department, Tor Vergata University of Rome, Rome, Italy

**Keywords:** autism, gene-environment interactions, epigenetics, neuroimaging, prospective studies, neurodevelopmental trajectories, tuberous sclerosis

In the last 50 years, research in Autism Spectrum Disorder (ASD) has constantly grown. However, etiology and pathogenesis of this disorder are still a matter of speculation. Thus, without reliable biomarkers ASD continues to be defined only by symptoms, being diagnosed by observing behavior and by asking questions to caregivers. As a result, established treatments for ASD core symptoms are still lacking. These limitations in knowledge are to some degree justified by the complexity and great heterogeneity of the ASD clinical phenotypes. Nevertheless, we maintain that recent developments in the understanding of gene-environment interactions are opening up new research outlooks, which might eventually lead to a leap forward in our comprehension and treatment of this disorder.

Throughout the twentieth century ASD research, like many other conditions, has been the victim of virulent debates about the role of environmental vs. genetic determinants of disease, which sometimes turned into ideological rather than scientific face-offs. Autism too has swung between the “solely environment” approach of Bettelheim age and the “solely genetic” attitude of more recent periods. However, today we are more aware than ever that the large majority, if not all, disease processes–as well as human differences–are determined by both genetics and the environment.

According to the current DSM-5 definition, the core symptoms of ASD consist in persistent deficits in social communication and social interaction across multiple contexts and in restricted, repetitive patterns of behavior, interests, or activities, which lead to clinically significant impairment in social, occupational, or other important areas of current functioning (1). These impairments, piling up, unfold in a large variability of clinical phenotypes. Some people present with intellectual disability, while others score above average on intelligence tests but struggle to communicate verbally or make compulsively repetitive movements. In some individuals, the disorder reveals itself only subtly so that a first diagnosis is made in adulthood (2). This wide diversity in ASD phenotypes has led researchers to link the disorder to sundry genetic and environmental risk factors, which might act in relation to each other.

In 2016, there were an estimated 62 million cases of ASD worldwide, accounting for a prevalence of 0.83% (3). In terms of disease burden, ASD accounted globally for more than 9 million Years Lived with Disability and for 121 Disability Adjusted Life Years per 100,000 population (4). In high-income countries, ASD prevalence has been estimated to be about 1% across all ages (2). However, according to a recently published US surveillance study, in 2014 the overall ASD prevalence in 8-year old children was 1.68% (5). This new estimate represents a 15% and a 150% increase over 2012 and 2000, respectively. Therefore, ASD prevalence in the US appears to have increased in the last decades, but the causes of this surge are not yet fully understood.

During the first decades of this century, developments in gene-hunting techniques identified several ASD associated genes, including genes that code for proteins involved in synaptic functions (6, 7). So far, no major causative gene has been isolated, but hundreds of risk genes have been suggested, with either highly penetrant rare variants or common variants with little effects. Some advancements in the detection of rare genetic variants have been made by studying genetic syndromes, which are known to contribute to the risk of ASD (8). As a matter of fact, the study of ASD that comes along with some genetic conditions–the “syndromic ASD”–is beginning to shed a new light on the pathophysiology of the disorder (9). ASD phenotypes are, e.g., more likely in individuals with tuberous sclerosis complex (TSC), Fragile X, Rett, or Angelman syndromes. By studying ASD associated with these diseases, some evidence is emerging that even heterogeneous ASD phenotypes might possibly present with a convergent pathophysiology, namely a dysregulation of intracellular pathways (6). Recently, some lessons have been learned from TSC, a disorder caused by a mutation in the TSC1 or TSC2 gene, which exhibits an overactivation of the mammalian target of rapamycin (mTOR) signaling pathway, with ensuing alteration of cell proliferation and differentiation accompanied by an increased risk of autism (10). Recent findings of our group showed how persistent seizures could have an additive effect, increasing the likelihood of autistic behaviors in infants with TSC (11). This condition has been used also as a model to investigate the role of the cerebellum in the pathogenesis of ASD (12). Overall, there is now the actual possibility that integrated genomic approaches, supported by advanced mathematical modeling, might lead to a better understanding of the pre and post-natal pathogenetic mechanisms of ASD and to the development of personalized molecularly targeted therapies (13). In addition, genome-wide association studies are increasingly considered an effective methodology to detect links between a common variant located in a particular DNA regions and the risk of developing ASD; however, the more a scrutinized variant is common, the more such studies require large sample sizes (14). Despite such meaningful headways, the identified variants–according to models–appear to be merely the initial ones of a much larger array (15). At any rate, the clinical heterogeneity of ASD seems to be related to a genetic heterogeneity as well.

On the other hand, even if in ASD the genetic component is substantial, a co-presence of several environmental factors has to be considered as well. Twin studies point to a heritability of the disorder, but also to a role of the environment (16). Possible environmental risk factors include advanced parents' age, pregnancy complications and maternal conditions, organic toxicants, air pollution, or medication exposure during pregnancy (17). Several of these factors can produce an effect on brain development in the course of pre and perinatal periods. Nonetheless, determining the causal pathway of environmental risk factors is challenging as they can act directly on the CNS or indirectly through other biological mechanisms. Cesarean section, e.g., is an alleged risk factor for several neurodevelopmental disorders, including ASD (18). Its effect might be direct or indirect by altering the characteristics of the intestinal microbiome of the newborn, which in turn could play a role in the development of autism (19).

When considering the role of the environment, it is likewise important to highlight that nowadays we recognize that also the pre-natal environment plays a role in the development of ASD. Increasing evidence, especially from large twin studies, shows that the uterine environment has an important impact on development, and that the mother's health can deeply influence the long-term mental and physical health of the developing embryo or fetus (20). In this perspective, ASD might be considered, at least in some cases, a disorder of fetal programming. While we are becoming more aware that some environmental factors wield their effect particularly during the pre-natal period–a crucial phase of life–there is the need to better appraise how genetics interacts with the environment in the womb. When pondering the extensive variability of ASD phenotypes, it is quite likely that it is related to an interplay of genetic, environmental, and uterine environmental factors (21). Researchers are actually increasingly considering a major role for multiple gene-environmental interactions, which might be one of the main causes of the wide inter-individual heterogeneity of ASD (16). However, such a multiplicity of factors makes it difficult to determine which one can be considered the primary cause in each case. All these influences are probably important in some way, but how important and how they interact are still open questions. Nonetheless, these complex interactions call for a possible pivotal role of epigenetic mechanisms, which can allow the environment to modulate gene expression and shape the clinical phenotype. Several observations seem to point toward the existence of epigenetic dysregulation in ASD, implying the possibility that some factors exert their effects through epigenetic alterations, which in some cases are already present in the newborns (22, 23). Nevertheless, such evidence is not conclusive. To further expand these essential research fields, there is the need to set up revamped well-designed community-based epidemiological studies.

Thus, not only the etiology, but also the pathogenesis of the disorder is still a matter of speculation; notwithstanding statistical associations, possible causal pathways have yet to be confirmed. Consequently, effective treatments for core ASD symptoms are so far unavailable (24). Actually, this state of things is comparable to that of several other psychiatric disorders (25).

Yet, in recent years advanced neuroimaging techniques, including diffusion tensor imaging and tractography (26), informed our understanding of possible brain correlates in people with ASD. Alterations of the cortical/subcortical architecture (27), as well as of the connectome (11, 28, 29) of people with ASD are beginning to be spotted. Several studies sustain now the hypothesis of an early and extensive abnormal brain development, where the required balance between growth and phased pruning seems to be muddled up (30). These atypical developmental trajectories, sustained by both enlarged brain volumes and abnormal connectivity (31, 32), might come about even before the presence of manifest clinical symptoms (33). The considerable amount of neuroimaging data produced to date seems to point toward an imbalance of functions requiring communications between distinct brain regions and intraregional circuitry (30). However, further research is needed to be better able to comprehend atypical developmental trajectories in ASD; these should include multicenter prospective studies starting during the pre-natal period as well as large pooled analyses of neuroimaging data.

As a whole, we deem that the existing data support the hypothesis that the mechanism underlying ASD etiology results from the effects of diverse gene-environment interactions, with possible cumulative or even multiplicative effects not only at individual level, but also through the generations. The accumulation of these interplaying factors might explain—on top of an extension of diagnostic criteria—the likely increase in ASD prevalence.

However, we believe that if the knowledge of the causes and treatment of ASD is not yet compelling, it might be due to some intertwined reasons. Firstly, genetic/environmental interactions—and thus epigenetic factors–were poorly understood until now, and therefore have been taken too little into account. Secondly, decennial trends have been approached with epidemiological instruments that were not sensitive enough to detect long-lasting events. Lastly, ASD has been often studied as a distinct nosological entity, and not as part of a continuum, which can embrace other neurodevelopmental disorders (34). In fact, the co-occurrence of different disorders seems to be the norm rather than the exception. Actually, ASD increasingly appears to be not a single disorder, but a blend of common core symptoms accompanied by a large variability of other symptoms.

These drawbacks call for the need of methodologies that can take into account the complexity and heterogeneity of ASD, aiming to detect the interplay of the diverse and multiple risk and protective factors associated with ASD. We believe that the gateway to disentangle such complex interactions is to set up large genetically-informed long-term prospective studies, so to be able to measure early environmental exposures in relation to developments over time and to their interactions with genetics. These are necessary because the turning-out of an ASD phenotype is a dynamic process, which often continues to develop after diagnosis. Likewise, the interactions between an individual and his/her environment are not static, but always on the go.

The ecological, cross-sectional, or case-control studies commonly used in these contexts bear several limitations. These can be overcome only by cohort studies, including multi-generational ones. Such studies, while being carried out, allow taking into account new genetic/epigenetic evidence, as well as data arising from cellular, computational, or animal model systems.

Advances in technologies and widespread databases are continuing to disentangle the genetic aspects of ASD. Now that we are beginning to enlighten some of the epigenetic aspects of ASD, all the possible environmental risk factors should be looked at also from the perspective of a potential interconnection with genetics, in order to examine possible causal pathways and whether and how genetics interacts with environment in the development of the different phenotypes. Epidemiological terminology and study designs have to increasingly consider innovative methodologies, which make use of newly developed biomedical informatics, so as to integrate clinical, environmental and genetic data, including the use of biobanks or integrated databases. Only the integration of genetic and epigenetic data will facilitate a better understanding of the molecular mechanisms involved in autism. In this framework, mold-breaking cohort studies can allow tracking disease trajectories, so as to connect subtle clinical changes with the genetic heterogeneity of the disorder.

In the last decades, we achieved crucial advances in ASD research thanks to detailed analyses of clinical features, neurological abnormalities, environmental factors and genetic characteristics. Innovative breakthroughs might possibly come from a novel synthesis of all these components.

## Author contributions

PC and LE both contributed to the design of this study, independently reviewed the literature and participated to the writing of the draft. They both approved the final submitted version.

### Conflict of interest statement

The authors declare that the research was conducted in the absence of any commercial or financial relationships that could be construed as a potential conflict of interest.
